# Comparison of syndromic versus laboratory-confirmed diagnosis of *Neisseria gonorrhoeae* and *Treponema palladium*, infections at the selected health centers in Addis Ababa, Ethiopia

**DOI:** 10.1186/s12978-022-01395-w

**Published:** 2022-04-02

**Authors:** Enaniye Ayalew, Surafel Fentaw, Semira Ebrahim, Elias Seyoum, Zerihun Woldesenbet, Mistire Wolde

**Affiliations:** 1 Department of Medical Laboratory Sciences, College of Health Sciences, Addis Ababa University, Addis Ababa, Ethiopia; 2 Department of Microbiology, Ethiopian Public Health Institute, Addis Ababa, Ethiopia; 3 Department of Microbiology, Yekatit 12 Hospital and Medical College, Addis Ababa, Ethiopia

**Keywords:** Sexually transmitted infection, Syndromic case management, Laboratory-based approach

## Abstract

**Background:**

Sexually transmitted infections (STIs) are major infectious diseases worldwide. Around one million people get STIs every day and among them a high burden of the diseases seen in Sub-Saharan African countries. In most developing countries, including Ethiopia, STIs are diagnosed only using syndromic methods, although there seems to be no consensus between syndromic and laboratory-based research.

**Objective:**

To compare the effectiveness of a syndromic versus laboratory-based approach in the diagnosis of sexually transmitted infections, especially *Neisseria gonorrhoeae* (NG) and *Treponema palladium (*TP*)*, infections among those attending a public health center in Addis Ababa, Ethiopia.

**Methods:**

a cross-sectional study was conducted from April 2019 to March 2020, at selected health centers STIs clinics in Addis Ababa, Ethiopia. A total of 325 study participants were involved. From each participant after having socio-demographic data, additional blood, urethral and vaginal discharge was collected. Then serological, Gram stain, culture, and biochemical tests were performed. SPSS version 23 was used to enter and analyze data. All relevant bodies provided ethical approval, and each study participant gave written informed consent.

**Results:**

Among the total participants 167 (51.4%) were males; 177 (54.5%) between ages of 26 and 35; and 178(54.8%) single. Of the total 325 NG, and 125 TP syndromic managed suspected cases, only 163 (50%) and 38 (30.4%) were laboratory- confirmed positive cases respectively. However, there was no statistically significant difference between NG and TP syndromic versus laboratory diagnostic confirmed cases (P-value > 0.005).

**Conclusion:**

The present study indicated that even if, there were no statistical differences between syndromic versus Laboratory diagnosis confirmed NG and TP cases, more than half of syndromic cases could not be confirmed by laboratory diagnosed tests. Thus, to strengthen the present findings, further large-scale studies are recommended.

## Plain language summary

Sexually transmitted infections (STIs) are the leading infectious diseases worldwide, and the majority of cases are recorded in developing countries, including Ethiopia. However, diagnosis of STIs in most developing countries done syndromic based, since the laboratory-based tests are relatively expensive, need skilled manpower, and time taking. Therefore regular assessments on how closely related STIs syndromic versus laboratory-based diagnosis should be done, for the proper management of the diseases. In this regard, the present study demonstrated that there was no statistically significant difference between syndromic versus laboratory-based diagnosis of some certain STIs infections; *Neisseria gonorrhoeae* (NG) and *Treponema palladium (*TP*),* in selected public health centers in Addis Ababa, Ethiopia. However, more than half of syndromic cases could not be confirmed by laboratory diagnosed tests. Thus, to narrow the gap between the two diagnostic approaches, regular training, updates, and similar studies need to cascade in the country. In the meantime, in other developing countries too, regular assessment on STIs syndromic versus laboratory-based diagnosis should be done, to control and prevent STIs infections worldwide.

## Introduction

The term "sexually transmitted diseases" (STDs) refers to the various clinical symptoms and diseases caused by pathogens spread through sexual activity. More than thirty viral, bacterial, and parasitic infections can be transmitted from person to person through sexual transmission, including vaginal, oral, and anal sex, and some of these, such as syphilis and HIV, can be transmitted from mother to fetus [1–4].

Gonorrheal is a bacterial disease, caused by *Neisseria gonorrhoeae* (NG). It is a gram-negative intracellular diplococcic, non-capsulated, and Oxidase-positive organism. It is transmitted during sexual intercourse; by attaching itself to the host via the Pilli, and infecting mucous membranes of the urethra, endo-cervix, pharynx, or rectum [2, 5].

NG left untreated, may lead to prostatitis and epididymitis in men, and into the pelvic areas in women. Thus the bacteria may result, in infertility, ectopic pregnancy, and chronic pelvic pain, as well as prostates, pharyngitis, and on rare occasions, fever, rash, and arthritis. During pregnancy, the organism can spread to their babies' eyes and cause gonococcal ophthalmia and serious conjunctivitis in newborns [6].

*Neisseria gonorrhoeae* diagnosis may be suggestive, presumptive, or definitive. As a result, we used a presumptive diagnosis approach in this research, and most of the time, NG becomes resistant to many antibiotics that were previously very effective to treat. The most common treatment for *Neisseria gonorrhoeae* is a single intramuscular injection of 250 mg Ceftriaxone, according to Center for Disease Control (CDC) guidelines [1].

Syphilis is caused by a microorganism called *Treponema palladium* (TP), which is a motile spirochete and helically coiled microorganism. Sexual contact is one way for *Treponema palladium* to spread. Syphilis is a chronic disorder that is divided into three phases based on clinical results, making recovery and follow-up easier. There are three types of syphilis infection: primary, secondary, and tertiary [1, 7, 8].

TP may also infect the central nervous system, causing neuro-syphilis at any point of the disease. Syphilis diagnosis may be conclusive using Darkfield microscopic examinations or presumptive while using Venereal Disease Research Laboratory (VDRL) and *Treponema palladium* Rapid test [1, 8–11].

In general, syndromic, and laboratory-based diagnoses are two forms of STI diagnosis approaches. Except in rare cases, laboratory diagnosis is the most accurate process. Laboratory diagnosis of STIs, due to requiring specialized testing equipment and skilled technicians, is costly, time-consuming, and findings may not be available at the time of the visit; in most health institutions mainly in developing ones, may result delay in the start of treatment [1, 8–11].

Thus, in certain developing countries, such as Ethiopia, the syndromic STI diagnosis is the only possible option. Since it is cost-effective, simple, and fast investigations, syndromic diagnosis of STIs is critical for immediate treatment. However, asymptomatic infections are impossible to detect because they are based on subjective judgment. Furthermore, this approach has the potential to lead to over diagnosis and care. Since syndromic diagnosing technique has a major impact on socioeconomic, individually as well as in the region, client morale, healthcare provider integrity, and can be exposed to drug resistance [2, 3, 12–25]. Therefore regular assessments and updates on how closely related Syndromic versus laboratory-based diagnosis of STI diagnosis so essential to provide quality medical services for the patients.

## Materials and methods

### Study area

This research was carried out in three health centers; Janmeda health center woreda 06, Arada health center woreda 10, and Gerji health center woreda 13, Addis Ababa, Ethiopia. The selected health centers are administered under the Addis Ababa Health Bureau in Addis Ababa, Ethiopia. The study sample was tested at the Ethiopian Public Health Institute's Clinical Bacteriology and Mycology National Reference Laboratory, which is ISO certified laboratory, in Addis Ababa, Ethiopia.

### Study design and period

A cross-sectional study was conducted to compare the Syndromic approach versus Laboratory-based approach on STI at a selected Health center in Addis Ababa, Ethiopia, from April 2019–March 2020.

### Source population

All clients who visited in selected health center outpatient department out patient department, (OPD) for medical service with a compliant of sign and symptoms of STI.

### Study population

All eligible clients who visited in selected health center OPD for STI according to WHO standard Syndromic approach STI management guideline.

### Inclusion and exclusion criteria

#### Inclusion criteria

Clients who visited the study site OPD and have a history or signs and symptoms of *Neisseria gonorrhoeae* and *Treponema palladium* and willing to participate in the study.

#### Exclusion criteria

Clients who have no signs and symptoms of *Neisseria gonorrhoeae* and *Treponema palladium* and patients having a history of taking antibiotics for the past 10 days are to be excluded.

### Study variables

During the study, the socio-demographic status of each study participant, such as age, sex, occupational status, marital status, and educational status was included. In addition, during study participant selection, Symptoms of STIs included abdominal pain, abnormal genital discharge, foul smell in the genital area, excessive genital secretions, swelling of lymph nodes in the genital area, itching in the genital area, burning pain on micturition, pain during intercourse, and genital ulcers were considered.

### Measurement and data collection

#### Sample size calculation and sampling method

In this study sample size was calculated by using single population proportion formula with a confidence interval of 95% and 5% marginal error by taking prevalence of the case (74.1%) from a study conducted at Gondar town hospital and health centers, Ethiopia [26]. And by adding 10%of calculated sample sizes for contingency, finally, the sample size was determined to be three hundred twenty-five. And convenience sampling method is used [1, 27].

#### Data collection procedure

After taking written informed consent from each study participant, Standard questionnaires were used for collecting socio-demographic data by trained Health care workers. For those clients not attending formal education and poor in reading and writing (here considered as illiterate), the data collectors read the consent form, and when the study participants agreed, signed on the consent form. After completion, it was cleared for completeness, unrecorded value, and unlikely response, and manually cleaned all Laboratory results were registered on the data collection sheet. Complete physical and clinical assessment was performed on all study participants as per the WHO guideline.

#### Sample collection

Appropriate ureteral and vaginal discharge and blood (serum) samples were collected by a trained clinical nurse. The ureteral and vaginal discharges were transported by Amies transport media to the analysis site following SOPs (adopted from ISO-15189 for sample management). Meanwhile, the blood (Serum) VDRL tests were done at the data collection site medical laboratory units, following the standard SOPS.

### Laboratory analysis

#### For isolation and identification of *Neisseria gonorrhoeae*

The transported specimen was inoculated on to modified Thayer Martin media, containing vancomycin, colistin, nystatin, and trimethoprim. Then it was incubated under 5–10% CO_2_ for 24–72 h, meanwhile, a parallel Gram stain was performed to look for polymorph nuclear leucocytes with Gram-negative intracellular diplococci microscopically. Then the isolates were identified as *Neisseria gonorrhoeae* by presumptive diagnosis method based on colony morphology, Gram staining, catalase test, Oxidase test, and superoxide test.

#### For rapid immune chromatographic syphilis tests

Serum was used to detect *Treponema palladium* by presumptive method Venereal Disease Research Laboratory and *Treponema palladium* Rapid test. The test procedure and result reading were performed according to the manufacturer’s instructions ad SOPs.

For both tests, IQC tests were performed with known negative and positive control samples [26].

### Data quality assurance

• Pre-tested standardized questionnaire was used for comprehensiveness, effectiveness, reliability, and validity.
• Training was given for data collectors on data collection procedures with the support of investigators and trained responsible bodies.
• Culture media sterility was ensured by incubating in 5% CO_2_ enriched wet atmosphere candle jar each batch of the prepared media at 37 °C for 24 h and the Performance of prepared media was checked by inoculating with control ATCC strains of *Neisseria gonorrhoeae.*
• Implementing standards based on quality Assurance measurements SOP available on investigation site throughout the whole process of data collection and Laboratory work
• SOPs were followed for pre-analytical, analytical, and post-analytical processes in the study site.

### Data analysis and interpretation

For data entry and analysis, SPSS (the Statistical Package for Social Sciences for Windows) version 23 statistical software was used. Bivariate analysis was used to determine socio-demographic characteristics versus *Neisseria gonorrhoeae, Treponema palladium* and percentage was calculated.

### Ethical considerations

The study was approved by the Research and Ethics Committee of the Addis Ababa University College of Health Sciences Department of Medical Laboratory Technology, Ethical clearance was obtained from Addis Ababa Public Health Research and Emergency Management Directorate. The study aim and details were explained to each study participant before obtaining their written consent for data and specimen collection, Confidentiality was maintained at all levels by using codes rather than names of the study participants and the involvement of participants in the study was a voluntary basis.

Participants were informed of their rights to resign from the study at any level of the study without any restriction if they were feeling uncomfortable in the study. Their results of all positive individuals were reported to the respective health facilities for better management of the patients.

## Result

### Socio-demographic characteristics

A total of 325 STI symptomatically diagnosed and treated patients were enrolled in the study, and of them 167 (51.4%) were males and the remaining 158 (48.6%) were females. The majority of the study participants were between the ages of 26 and 35. (54.5 %). According to the marital status analysis, the majority of people were single 178 (54.8%); whereas only 19 (5.8%) were illiterate. In addition, 221 (68.1%) were non-healthcare practitioners, and 8 (2.5%) were commercial sex workers, as shown in Table 1.

**Table 1:** Frequency and percent of socio-demographic characteristics of study participants at selected health centers in Addis Ababa Ethiopia from April 2019–March 2020

Variable		Frequency	Percent
Age	18–25	101	31.1
	26–35	177	54.5
	> 35	47	14.5
Educational status	Illiterate	19	5.8
	Literate	306	94.2
Marital status	Single	178	54.8
	Married	135	41.5
	Divorced	11	3.4
	Widowed	1	0.3
Gender	Male	167	51.4
	Female	158	48.6
Current profession	Health profession	1	0.3
	Other professions	221	68.0
	Commercial sex worker	8	2.5
	Merchant	32	9.8
	Long-distance driver	6	1.8
	Jobless	23	7.1
	Others	34	10.5

In the present study, among the 325 syndromic *Neisseria gonorrhoeae* suspected study participants, only 163 (50 %) were laboratory-confirmed positive cases; whereas among 125 syndromic *Treponema palladium* suspected study participants, only 38 (30.4 %) were laboratory-confirmed positive cases, as shown in Table 2. Among the 163 laboratory-confirmed NG positive cases, 126 were male, and 37 females; meanwhile among 38 laboratory-confirmed TP positive cases, 26 were male and 12 females.

**Table 2:** Syndromic approach versus laboratory-based approach for *Neisseria gonorrhoeae* and *Treponema palladium* at selected health centers in Addis Ababa Ethiopia from April 2019–March 2020

Variables	Syndromic diagnose	Laboratory confirmed tests				P value
		Microscopic test	Culture test	Biochemical test	Serology test	
*N. gonorrhoeae*	325	163 (50.2%)	163 (50.2%)	163 (50.2%)	0	0.36
*T. Pallidum*	125	0	0	0	38 (30.4)	0.86

Although there was no statistical difference between NG and TP syndromic versus laboratory diagnostic confirmed cases (P-value > 0.005), the present study showed big differences seen between the two STIs diagnostic approaches in the selected health centers in Addis Ababa, Ethiopia (Table 2).

## Discussion

The main aim of our study was to evaluate syndromic versus laboratory-based diagnosis of STIs, mainly of NG and TP. Many developing countries, including Ethiopia, have adopted a syndromic approach to STI diagnosis and care. Studies indicated that the syndromic method may not be the most successful way to diagnose STI patients. Besides, obtaining the exact magnitude of STIs in most countries takes a lot of effort, due to factors, including stigma, lack of diagnostic facilities, and budget [20–23].

Our study indicated that among syndromic diagnosed 325 cases163 (50.2%) NG laboratory-confirmed, and from 125 TP cases 38 (30.4%) were laboratory-confirmed. A similar community-based syndromic versus laboratory diagnosis of sexually transmitted infections study conducted in Northern Tanzania, indicated that of the total participated 1520 women, 50% were positive for at least one STI, and of them, syphilis cases account 2.5% and gonorrhea 0.2% [28]. Another study conducted in India indicated that out of 1120 Syndromic STI cases, only 409 (36.5%) cases were confirmed in the laboratory for pathogens [23].

In our study, the laboratory-confirmed NG and TP cases were relatively higher than the compared two studies. These may be due to the laboratory diagnostic methods, distributions of STIs in different areas, study participants category (in our case both male and female, whereas only females in Tanzania). and our study focused only on NG ad TP specific STIs.

In the present study, the laboratory-confirmed NG cases were 163/325 (50.2%), with distributions of 126 (38.8%) cases male, and 37 (11.4%) cases female. Another study done in Addis Ababa, Ethiopia, showed that 7out of 58 (12.07%) urethral discharges were caused by NG [5]. In addition, a study conducted in Gondar town hospitals and health centers (Ethiopia) showed that out of 120 patients who tested positive for STIs, 89 (74.1%) were confirmed cases, and of them, 25 (20.8%) NG laboratory-confirmed ones [26].

On the other hand, our study indicated that from syndromic managed 125 TP cases, 38 (30.4%) were laboratory confirmed. Among the confirmed cases, 26 were male and 12 were females. A similar study done in India showed that out of 1120 Syndromic STI cases 2 cases (0.18%) were serologically confirmed syphilis [23]. A study done in Addis Ababa, Ethiopia, showed that four out of eight (50 %) genital ulcers were caused by syphilis [5]. A similar study conducted in Gondar town hospitals and health centers (Ethiopia) showed that out of 120 patients who tested positive for STIs, 89 (74.1%) were confirmed with TP [26].

In our study, there were no statistically significant differences seen between syndromic diagnosed NG and TP cases versus laboratory-confirmed ones (P > 0.005). A similar study conducted in northern Tanzania also indicated no statistical significance differences between patient-reported STIs symptoms and laboratory-confirmed STI tests [28]. However, large numbers of syndromic cases, in our case 49.8% of NG and 62% of TP did not confirm by the laboratory tests; and more than the number of our percentages in Tanzania similar research [28].

In most sub-Saharan African countries, STIs, including HIV is still high. Thus, as one of the control mechanisms of HIV epidemics in the region and elsewhere, as much as possible accurate laboratory-based diagnoses of STIs are so important. As disadvantages of syndromic STI diagnosis include missing of asymptomatic cases, such as NG cases [29] variations of the algorithm from country to country [30]. Thus, whenever possible, if STIs diagnosis is supported by laboratory tests, it will improve the quality of medical service and great support in STIs prevention and control strategies.

### Limitations

- There was a test specificity limitation for *Treponema palladium*
- Did not include other STI-causing pathogenic organisms.

## Conclusion and recommendation

### Conclusion

The present study indicated that there were no statistically significant differences between syndromic versus laboratory diagnosis confirmed NG and TP cases. However, there were more than half of syndromic cases could not be confirmed by laboratory diagnosed tests.

### Recommendation

The syndromic approach for STI cases in developing countries is important, to give a quick diagnosis ad treatment of patients. However, as the laboratory method is a golden diagnostic standard, the closeness of Syndromic diagnostic methods with the laboratory-confirmed cases should be done regularly. Furthermore, to strengthen the present findings, similar large-scale studies are strongly recommended, both inland, and internationally.

## Data Availability

All necessary data related to the research presented within the article. However, upon request, corresponding author can provide any additional data if needed.

## Abbreviations

CDC: Center for Disease Control
NG: *Neisseria gonorrhoeae*
OPD: Out patient department
STDs: Sexually transmitted diseases
STI: Sexually transmitted infection
STIs: Sexually transmitted infections
TP: *Treponema palladium*
VDRL: Venereal Disease Research Laboratory
