# Correction to: Comparison of syndromic versus laboratory-confirmed diagnosis of *Neisseria gonorrhoeae* and *Treponema pallidum*, infections at the selected health centers in Addis Ababa, Ethiopia

**DOI:** 10.1186/s12978-022-01416-8

**Published:** 2022-05-05

**Authors:** Enaniye Ayalew, Surafel Fentaw, Semira Ebrahim, Elias Seyoum, Zerihun Woldesenbet, Mistire Wolde

**Affiliations:** 1 Department of Medical Laboratory Sciences, College of Health Sciences, Addis Ababa University, Addis Ababa, Ethiopia; 2 Department of Microbiology, Ethiopian Public Health Institute, Addis Ababa, Ethiopia; 3 Department of Microbiology, Yekatit 12 Hospital and Medical College, Addis Ababa, Ethiopia

## Correction to: Reproductive Health (2022) 19:88 https://doi.org/10.1186/s12978-022-01395-w

After publication of this article [1], it was reported that the term '*Treponema palladium*' should have read '*Treponema pallidum*'.

The original article [1] has been updated.
